# Exposure of Biomimetic Composite Materials to Acidic Challenges: Influence on Flexural Resistance and Elastic Modulus

**DOI:** 10.3390/biomimetics5040056

**Published:** 2020-10-28

**Authors:** Andrea Scribante, Simone Gallo, Stefano Scarantino, Alberto Dagna, Claudio Poggio, Marco Colombo

**Affiliations:** Department of Clinical, Surgical, Diagnostic and Paediatric Sciences—Section of Dentistry, University of Pavia, Piazzale Golgi 2, 27100 Pavia, Italy; stefano.scarantino01@universitadipavia.it (S.S.); alberto.dagna@unipv.it (A.D.); claudio.poggio@unipv.it (C.P.); marco.colombo@unipv.it (M.C.)

**Keywords:** dentistry, conservative, restorative, materials, nanohybrid, resin composites, ormocer-based composites, acidic drink, acid, flexural strength, elastic modulus

## Abstract

Acidic conditions of the oral cavity, including soft drinks and cariogenic bacteria, represent a damage for restorative biomimetic composite materials. The aim of this study is to assess the influence of two different acidic challenges on the flexural strength and elastic modulus of five composites: x-tra fil (Group 1, XTF), GrandioSO x-tra (Group 2, GXT), Admira Fusion x-tra (Group 3, AFX), VisCalor bulk (Group 4, VCB), and Enamel Plus HRi (Group 5, EPH). Thirty samples for each group were randomly divided and assigned to three different treatments: storage in distilled water as the controls (subgroups 1a–5a), 3 weeks distilled water + 1 week Coca-Cola (subgroups 1b-5b), and 4 weeks Coca-Cola (subgroups 1c–5c). For each subgroup, the flexural strength and elastic modulus were measured using an Instron universal testing machine, and data were submitted to statistical analysis. Considering subgroups B, no material showed a significant difference in the flexural strength with the controls (*p* > 0.05), whereas for subgroups C, only GXT and VCB showed significantly lower values (*p* < 0.05). AFX reported the lowest flexural strength among the materials tested. As regards the elastic modulus, no material showed a significant variation after acidic storages when compared with the respective control (*p* > 0.05). AFX and EPH reported the lowest elastic modulus compared to the other materials. All composites tested showed adequate flexural properties according to the standards, except for AFX. This biomimetic material, along with EPH, might be indicated for V class (cervical) restorations considering the lowest values of elasticity reported.

## 1. Introduction

Along with periodontitis, dental caries represents the most common oral disease and the major cause of tooth loss [1]. It consists of an infective process leading to the dissolution of hydroxyapatite, the mineral component of the hard tissues of tooth and bone [2]. In particular, orthodontic patients might be more susceptible to demineralization around brackets and bands, due to plaque accumulation [3]. However, non-carious lesions might also affect the integrity of dental elements and, within this group, erosions, abrasions, attrition, and abfractions are included [4]. During dental erosion, the same fade of the tooth’s hydroxyapatite occurs as reported for decay, this time without the action of acidogenic bacteria but following the exposition to both intrinsic (e.g., gastroesophageal reflux, eating disorders associated with vomiting) or extrinsic (e.g., acidic drinks, bleaching procedures) acidic factors [5,6]. Focusing on acid drinks, they have become more and more popular in recent years, especially among the young. For example, it has been shown that between 56% and 85% of children daily consume such beverages [7]. The demineralizing action of acids on the teeth consists of an attack exerted by hydrogen ions (H^+^) against the anions of the enamel crystals (CO_3_^2−^ e PO_4_^3−^) with a consequent erosion. If remineralization does not occur by means of saliva or a remineralizing agent, a definitive loss of the outer enamel takes place, while the layer below becomes softer [8]. In addition to the effect on the dental structure, these acid substances might also have consequences on general health considering, for instance, the major risk of gastritis to which consumers are exposed, along with the risk of overweight, obesity, and type 2 diabetes [9]. 

With the aim of restoring the dental structure, the infected dental tissue, if present, must be completely removed and cavities generally filled with biomimetic composites in order to simulate the anatomical condition [10]. Considering the masticatory loads, especially in the posterior segment of the dental arches, restorative materials should guarantee good mechanical properties and much research has been conducted in this regard [11,12,13]. However, even restorative materials are subjected to the action of acids of the oral cavity with a corrosion and a subsequent alteration of their initial characteristics. For instance, in vitro microhardness has been shown to be significantly decreased after acidic exposure because of a degradation of the polymer network of the composite and the falling out of the resin, with an eventual risk of secondary decay [14]. 

Properties such as flexural strength have been largely investigated in orthodontics as regards fiber-reinforced composites retainers both in vitro [15,16,17,18] and clinically [19,20]. Conversely, to date, fewer studies have been reported that assess the variation of these parameters after exposure to acidic beverages [21,22,23]. 

Accordingly, the purpose of the present research is to assess the flexural strength and elastic modulus of common restorative composites after different acidic storages. The null hypothesis is that for each material tested there is no significant difference in the flexural strength and elastic modulus for the five different composite resins neither after a three-week acid challenge (followed by a one-week storage in distilled water) nor after a four-week acid challenge when compared to controls stored in distilled water during the whole experimentation.

## 2. Materials and Methods

### 2.1. Specimen Preparation

Five different composites were considered for this study and subdivided into respective groups: x-tra fil (Voco, Cuxhaven, Germany) (Group 1, XTF), GrandioSO x-tra (Voco, Cuxhaven, Germany) (Group 2, GXT), Admira Fusion x-tra (Voco, Cuxhaven, Germany) (Group 3, AFX), VisCalor bulk (Voco, Cuxhaven, Germany) (Group 4, VCB), and Enamel Plus HRi (Micerium, Genova, Italy) (Group 5, EPH). The characteristics of the materials tested are shown in Table 1.

Thirty samples were considered for each group, a sample calculation test was performed using the software Sample Size Calculator (Calculator.net, 2203 Timberloch PI, Suite 252, The Woodlands, TX 77380, USA) [24].

Each material was inserted into thirty rectangular prism-shaped stainless-steel molds (25 mm length × 2 mm depth × 2 mm height), with a surface-to-volume ratio of 2.08 mm^−1^ [25]. Before its application, the VisCalor bulk was preheated with the preheating device (Caps Warmer, Voco, Cuxhaven, Germany) at 68 °C for 15 min, in accordance with the operating instructions, due to its thermoviscous behavior.

Each mold was completely filled, and a polyester matrix strip (Mylar strip, Henry Schein, Melville, NY, USA) was positioned above to form a flat surface and to prevent oxygen from interfering with the polymerization of the most superficial layer of the composite [26]. Samples were then photopolymerized for 3 min [16] into a light-curing oven (Spectramat, Ivoclar Vivadent AG, Schaan, Liechtenstein) with a light intensity of 1200 mW/cm^2^, a wavelength of 430–480 nm, lamp socket R7s, a lamp diameter of 13.5 mm, and a lamp length of 160 mm. 

The bars of composite obtained were removed from the molds and stored in water for 48 h in the dark, at 37 °C and 100% humidity [27].

Subsequently, the thirty specimens of the 5 groups were randomly divided into three subgroups (A, B, and C) of 10 specimens each, with an assigned storage for each one as here listed:

(1) subgroups A (1a–5a): 4-week storage in 50 mL distilled water (control subgroups);
(2) subgroups B (1b–5b): 3-week storage in 50 mL distilled water + 1-week storage in 50 mL soft drink;
(3) subgroups C (1c–5c): 4-week storage in 50 mL soft drink.

The times of the acid challenges are based on a previous study reported in the literature considering 1 and 4 weeks in soft drinks [28].

Both distilled water and the soft drink used (Coca-Cola, Coca-Cola Company, Milano, Italy) were at room temperature (18 ± 1 °C). Specimens were immersed singularly in the 50 mL solutions, and in the case of subgroups C, the acidic drink was changed weekly [29]. This solution had a pH value of 2.52 which was measured before each immersion of the specimens. Moreover, it was checked before testing in both subgroups B and C. No remarkable variations of the pH value occurred during the various measurements. 

### 2.2. Three-Point Flexural Test

**Figure 1:**
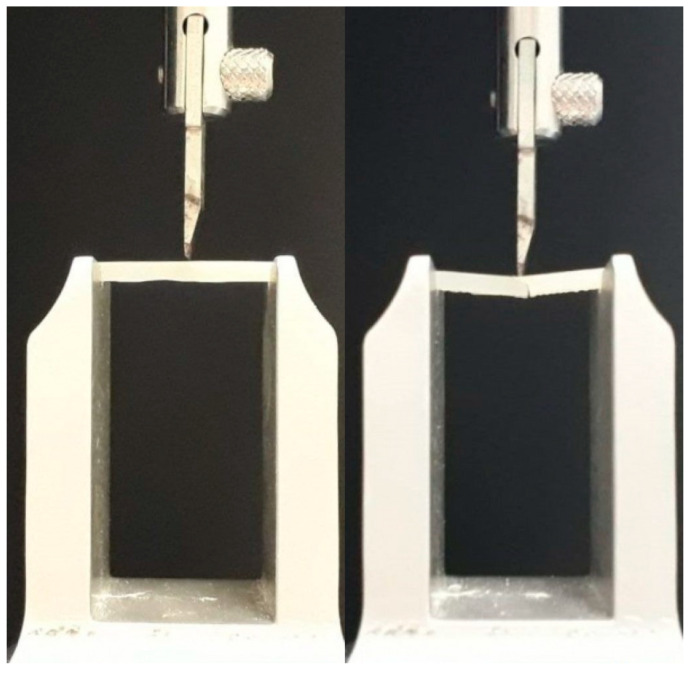
Mechanical tests performed on the samples (span length: 21mm): left, sample before the fracture; right, sample at the moment of the fracture.

Each sample was positioned inside an aluminum support having a distance of 21 mm between the two arms. A universal testing machine (Model 3343, Instron Corporation, Canton, MA, USA) was used to apply a compressive load on the middle of the specimens with a crosshead speed of 1.0 mm per minute until the failure (Figure 1) [30]. Flexural strength (*σ*) and elastic modulus (E) were calculated as follows [31]:

σ = 3FL/(2BH^2^) (1)

where F is the maximum load (Newtons), L is the distance between the arms (millimeters), B is the width of the samples (millimeters), and H is the height (millimeters)

E = FL^3^/4BH^3^d (2)

where F is the maximum load, L is the distance between the arms, B is the width of the samples, H is the height of the specimen, and d is the deflection (in millimeters) corresponding to the load F.

Statistical analyses were performed with computer software (R version 3.1.3, R Development Core Team, R Foundation for Statistical Computing, Wien, Austria). Descriptive statistics were calculated (mean, standard deviation, and the minimum and maximum value). The normality of the distributions was assessed with the Kolmogorov and Smirnov test. Nonparametric analysis of variance (Kruskal–Wallis method) was applied to determine the presence of significant differences among the various groups considered [32]. The Mann–Whitney post hoc test was applied. Significance for all statistical tests was predetermined at *p* < 0.05.

## 3. Results 

### 3.1. Flexural Strength

**Figure 2:**
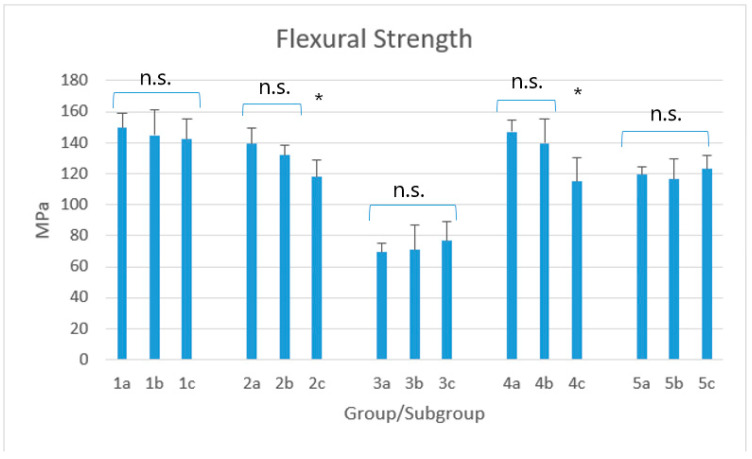
Mean flexural strength (MPa) for each group/subgroup. Legend: 1: x-tra fil; 2: GrandioSO x-tra; 3: Admira Fusion x-tra; 4: VisCalor bulk; 5: Enamel Plus HRi. Subgroups A (1a–5a): 4-week storage in 50 mL distilled water (control subgroups); Subgroups B (1b–5b): 3-week storage in 50 mL distilled water + 1-week storage in 50 mL soft drink; Subgroups C (1c–5c): 4-week storage in 50 ml soft drink. n.s.; non-significant; *: asterisks indicate the presence of significant differences among the subgroups.

Descriptive statistics of the various groups are shown in Table 2 and Figure 2. 

The Kruskal–Wallis method showed significant differences among groups (*p* < 0.0001). Post-hoc showed that for groups 1, 3, and 5, a statistical difference occurred among the corresponding subgroups (*p* < 0.05) but not within the three subgroups of each material (*p* > 0.05). Subgroups 2a and 2b as well as subgroups 4a and 4b showed no significant difference with group 1 (*p* > 0.05) but, conversely, subgroups 2c and 4c showed significant lower values (*p* < 0.05), comparable with those assessed in group 5 (*p* > 0.05). 

### 3.2. Elastic Modulus

**Figure 3:**
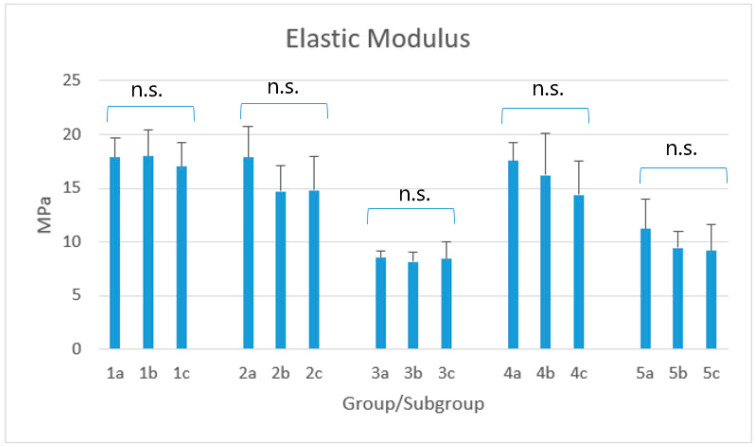
Mean elastic modulus (MPa) for each group/subgroup. Legend: 1: x-tra fil; 2: GrandioSO x-tra; 3: Admira Fusion x-tra; 4: VisCalor bulk; 5: Enamel Plus HRi. Subgroups A (1a–5a): 4-week storage in 50 mL distilled water (control subgroups); Subgroups B (1b–5b): 3-week storage in 50 mL distilled water + 1-week storage in 50 mL soft drink; Subgroups C (1c–5c): 4-week storage in 50 mL soft drink. n.s.; non-significant; The Kruskal–Wallis method showed significant differences among groups (*p* < 0.0001). Post-hoc showed that the highest elastic modulus was reported for groups 1, 2, and 4, with no statistical difference neither among the corresponding subgroups (*p* > 0.05) nor among the three subgroups of each material (*p* > 0.05). Values reported for groups 3 and 5 were significantly lower (*p* < 0.05) but, even this time, without significant differences neither among the corresponding subgroups, nor among the three subgroups of the two materials (*p* > 0.05). Finally, the values of subgroups 4c and 5a showed no statistical difference among them (*p* > 0.05).

Descriptive statistics of the various groups are shown in Table 3 and Figure 3. 

## 4. Discussion

In the field of restorative dentistry, biomimetic composite materials have been undergoing a rapid evolution considering the number of products proposed. These are extremely more appreciated if compared to other ones previously proposed, like amalgam [33]. However, the acidic conditions of the oral cavity, such as the consumption of soft drinks or acidic foods and the presence of acidophile bacteria, constitute a danger not only for teeth but even for filling materials [34]. In particular, a recent study considering four of the five products tested in the present report (x-tra fil, GrandioSO x-tra, Admira Fusion x-tra, VisCalor bulk) stated that their microhardness is significantly reduced when stored in an acidic beverage, both for 1 day and for 1 week, with respect to the samples kept in water [14]. In the literature it has been supposed that, when exposed to low pH conditions, the filler of the resin tends to fall out and the matrix tends to decompose [35]. The dissolution of both enamel and restorative dental composites has been shown to take place under a pH value of 4 [36].

Among the mechanical properties, the flexural strength and elastic modulus have been studied until now in orthodontics [37], endodontics [38], and prosthodontics [39], particularly for fiber-reinforced composites. The flexural strength is a relevant index to identify the capacity to support masticatory loads, whereas the elastic modulus guides clinicians to choose the right material for the specific clinical case. Despite previous reports that have been conducted on nonfiber-reinforced composites, the variation of the abovementioned parameters after immersion into acidic solutions has not been widely investigated. Accordingly, the purpose of the present report was to evaluate the influence of different acidic storages on various resin composites and on an ormocer-based material commonly used in a clinical setting.

The first null hypothesis of this study has been partially accepted. No significant difference has been detected for XTF, AFX, and EPH (*p* > 0.05), independently of the acidic challenge (3 weeks in water + 1 week in Coca-Cola vs 4 weeks in Coca-Cola) and with respect to the controls stored in water for 4 weeks. Conversely, both GXT and VCB showed a significant difference with the controls when storing samples only in Coca-Cola, but not when the acidic challenge was preceded by 3 weeks in distilled water. Therefore, the erosive action of Coca-Cola exerted for 1 week did not significantly affect the flexural strength. Conversely, the 1-month acidic challenge was able to significantly decrease this parameter for only two materials tested, which was not dependent of the percentage of their filler content, considering that it was similar with that of other materials that did not report a significant variation. As well, it was not dependent on the fact that these two materials are bulk-fill composites, since some of the other materials not showing the abovementioned behavior belong to bulk-fill category as well. Therefore, this might be justified by the different concentrations of the chemical components which are not disclosed by the manufacturers. 

On the basis of the results obtained during this first part of the experimentation, it should be emphasized that, however, standard deviations of the mean values of the flexural strength for each group/subgroups were almost always higher than 5%, which is the maximum value according to ISO 178/2010 [40].

As regards subgroups A and B, it is not possible to establish whether the immersion into distilled water might have altered the flexural strength, since no comparison has been carried out with dried controls. Contradictory results have been reported in the literature [41,42].

In the only previous study dealing with this topic, the nanofilled composite Filtek Supreme XTE, despite the highest initial values, was the only one to show a statistical reduction in the flexural strength when comparing the one-week storage in Cola with the controls kept in water for 24 hours; on the contrary, the other materials tested did not significantly vary their flexural properties, even after 1 month in acidic drink [25]. These outcomes partially disagree with the results obtained for the materials tested in this report, since we have not even found a material reporting a significant decrease in flexural strength after one week. Conversely, we stated that there were two composites whose values were significantly lower after the 1-month acid challenge.

Admira Fusion x-tra is the material reporting the lowest value of the flexural strength. Similarly, the previous study assessed an analogue characteristic for Admira Fusion [25]. Moreover, in another report, Admira Fusion x-tra was the one reporting the lowest microhardness value of both the external and internal sites after polymerization, as well as the highest mean percentage reduction in microhardness after acidic storage for both 1 day and 1 week in Coca Cola [14]. Both Admira Fusion and Admira Fusion x-tra belongs to a particular group of materials which differ from the other ones tested. In fact, they are based on the technology called Ormocer, an acronym of “organically modified cermics”, which consists of inorganic-organic co-polymers in addition to the inorganic silanated filler particles [43]. Ormocers have improved biocompatibility compared to resin-based restorative dental materials [44] but, according to the systematic review and meta-analysis of Monsarrat et al., [45] the first generation of ormocers shows a worse clinical behavior than conventional composites, in particular after long-term aging. The ISO 4049/2009 [46], subsequently revised by The Academy of Dental Materials [47], establishes a minimum value of 80 MPa to consider polymer-based restorative materials adequate for filling occlusal surfaces. Neither Admira Fusion x-tra in the present report nor Admira Fusion in the previous one has exceeded this ideal value, not even the control samples. Conversely, according to our results, all the other materials tested were largely above this limit, independently of the storage condition, and therefore they might represent adequate materials for high-stress bearing areas.

The second null-hypothesis has been accepted. In fact, for none of the materials tested a variation of the elastic modulus occurred after the two different acidic storages if compared to controls, which is in accordance with the previous study mentioned [25]. The highest values of elastic modulus were reported for x-tra fil, GrandioSO x-tra and Viscalor bulk, with no significant difference among them. On the opposite, both Admira Fusion x-tra and Enamel Plus HRi showed significantly lower values. This latter had the lowest percentage of filler content which justify the low values of flexural strength reported in the three different conditions: an increase of filler content has been shown to be related to higher values of elastic modulus [48].

It might be deduced that Admira Fusion x-tra and Enamel Plus HRi are more reliable when used for V class (cervical) restorations, since their higher elasticity can absorb the indirect stresses generating at the cervical zone of the tooth [25]. Conversely, the higher elastic modulus of x-tra fil, GrandioSO x-tra and VisCalor bulk might justify their use in occlusal areas requiring stiffer composites.

In a previous study, the materials corresponding to groups 1 to 4, after being cured for 20 seconds with a LED unit having an output irradiance of 1000 mW/cm^2^, have shown an adequate depth of cure represented by a hardness ratio greater than 0.80 [14]. Since the conditions of photopolymerization considered in the present report appear to be more extreme (3 min into a light-curing oven with a light intensity of 1200 mW/cm^2^), it can be supposed that the leaching of monomers into the media with the activation of enzymatic degradation probably did not occur and therefore there is no alteration of the effect caused by the acidic environment on the mechanical parameters here considered.

The main limitation of this report is that it has been conducted in vitro, therefore the buffering capacity of saliva, which contrasts the erosive action of acids [49], has not been considered. Controls were not stored in a dry environment but in distilled water which might have partially influenced the parameters studied. Actually, a previous study found no significant alteration of the flexural strength for composites stored in distilled water for 7 days when compared to the controls [41]. However, in our report, the samples of subgroups A were kept in water for 4 weeks, which is a longer time and an alteration of the parameters studied might have really occurred. Therefore, it would be interesting to further confirm our preliminary results by measuring not only flexural strength and elastic modulus after storage in water (positive controls), but even before (negative controls). As well, considering the eventual action exerted by distilled water, the experimentation should also provide a weekly change of the medium, as done in this study for the acid solution.

As regards the acidic challenges, the experimentation was conducted under extreme conditions represented by storages in Coca-Cola for an entire week (after 3 in water) and for 4 weeks. However, this continuous exposure to soft drink simulates a long-term exposure in the oral cavity: immersion in Coca-Cola for 1 day is comparable to an in vivo exposure for a month [50]. Moreover, chemical erosion might change the physical dimensions of the exposed samples, therefore this variation should be considered to correct the calculations of both the flexural strength and elastic modulus. Finally, it would be of interest to measure further parameters besides the two here considered, such as the surface roughness at the tension side of the beam detecting any correlation with the strength decrease, as well as to complement the results with images from optical and SEM microscopy. Further in vivo studies are required to confirm the results obtained, and other biomimetic restorative materials such as compomers and glass ionomers should be tested because of their higher susceptibility to an acidic storage media, due to a buffering action exerted towards it [51].

## 5. Conclusions

Under the limitations of this in vitro study, we can conclude that the flexural strength was significantly affected only for GXT and VCV after storage in Coca-Cola for an entire month. None of the different acidic exposures considered in this study have significantly altered the elastic modulus of the biomimetic materials tested.

## Figures and Tables

**Table 1:** Characteristics of the materials tested.

Group	Material	Code	Type	Composition	Filler Content %	Lot Number	Manufacturer
1	x-tra fil	XTF	Light-curing posterior filling material	*Matrix*: dimethacrylate (Bis-GMA, TEGDMA, UDMA) *Filler*: Inorganic filler (Bariumaluminium silicate, fumed silica, pigments)	86 (*w/w*)	1906144 Ex: 08/2021	Voco, Cuxhaven, Germany
2	GrandioSO x-tra	GXT	Aesthetic nanohybrid bulk restorative material	*Matrix*: Bis-GMA, Bis-EMA, aliphatic dimethacrylate *Filler*: Inorganic filler, organically modified silica	86 (*w/w*)	1907626 Ex: 08/2020	Voco, Cuxhaven, Germany
3	Admira Fusion x-tra	AFX	Nano-hybrid ORMOCER^®^-based material	*Matrix*: ORMOCER^®^ *Filler*: glass ceramics, silica nanoparticles, pigments	84 (*w/w*)	1904427 Ex: 04/2021	Voco, Cuxhaven, Germany
4	VisCalor bulk	VCB	Termoviscous bulk-fill composite (Nano-hybrid composite)	*Matrix*: Bis-GMA, aliphatic dimethacrylate *Filler*: Inorganic filler	83 (*w/w*)	76292 Ex: 06/2019	Voco, Cuxhaven, Germany
5	Enamel Plus HRi	EPH	Nano-hybrid composite	*Matrix*: Diurethandimethacrylate, BisGMA, 1,4-butandioldimethacrylate *Filler:* surface-treated nano zirconium oxide particles, glass	74% (*w/w*)	2018004910 Ex: 07/2021	Micerium, Genova, Italy

**Table 2:** Descriptive statistics of flexural strength (MPa) for each group/subgroup.

Material Code	Group-Subgroup	Mean (*)	Standard Deviation (%)	Minimum	Median	Maximum
XTF	1*a*	149.66 ^a^	6.18	132.30	150.22	166.95
XTF	1*b*	144.78 ^a^	11.22	123.24	143.13	172.86
XTF	1*c*	142.18 ^a^	9.26	123.24	141.16	172.46
GXT	2*a*	139.31 ^a^	7.48	122.06	138.60	154.74
GXT	2*b*	131.91 ^a^	4.73	121.67	131.71	140.18
GXT	2*c*	118.09 ^b^	8.84	100.80	120.09	129.94
AFX	3*a*	69.58 ^c^	7.62	59.06	70.68	75.99
AFX	3*b*	70.98 ^c^	22.57	43.71	71.86	101.59
AFX	3*c*	76.66 ^c^	16.11	56.31	78.95	95.68
VCB	4*a*	147.18 ^a^	4.82	135.06	147.07	159.47
VCB	4*b*	139.55 ^a^	11.54	109.07	138.21	163.80
VCB	4*c*	115.01 ^b^	13.28	91.35	117.14	137.42
EPH	5*a*	119.31 ^b^	4.30	111.43	119.70	128.36
EPH	5*b*	116.55 ^b^	11.36	92.93	116.55	137.81
EPH	5*c*	123.18 ^b^	6.83	111.43	121.67	135.06

(*) Superscript letters (a, b and c) have been used to indicate statistical results: different letters indicate the presence of significant differences in flexural strength among the groups.

**Table 3:** Descriptive statistics of elastic modulus (MPa) for each group/subgroup.

Material Code	Group-Subgroup	Mean (*)	Standard Deviation (%)	Minimum	Median	Maximum
XTF	1*a*	17.87 ^a^	10.12	13.89	18.23	20.45
XTF	1*b*	17.99 ^a^	13.56	15.09	17.53	22.65
XTF	1*c*	17.01 ^a^	13.35	12.37	17.29	21.13
GXT	2*a*	17.90 ^a^	15.70	14.95	17.39	25.03
GXT	2*b*	14.69 ^a^	16.54	9.64	15.41	17.17
GXT	2*c*	14.83 ^a^	37.58	11.94	14.52	22.86
AFX	3*a*	8.52 ^b^	7.63	7.24	8.66	9.31
AFX	3*b*	8.16 ^b^	11.15	7.04	7.96	9.41
AFX	3*c*	8.42 ^b^	19.48	5.68	8.33	10.76
VCB	4*a*	17.58 ^a^	9.73	13.39	18.02	19.53
VCB	4*b*	16.23 ^a^	23.66	12.04	15.77	25.54
VCB	4*c*	14.40 ^a,c^	21.53	11.19	13.77	21.92
EPH	5*a*	11.20 ^b,c^	25.00	6.35	11.27	14.90
EPH	5*b*	9.45 ^b^	16.51	6.33	9.77	11.29
EPH	5*c*	9.16 ^b^	26.97	6.20	9.47	12.17

(*) Superscript letters (a, b and c) have been used to indicate statistical results: different letters indicate the presence of significant differences in elastic modulus among the groups.
